# TRAIL sensitize MDR cells to MDR-related drugs by down-regulation of P-glycoprotein through inhibition of DNA-PKcs/Akt/GSK-3β pathway and activation of caspases

**DOI:** 10.1186/1476-4598-9-199

**Published:** 2010-07-28

**Authors:** Suk-Bin Seo, Jung-Gu Hur, Mi-Ju Kim, Jae-Won Lee, Hak-Bong Kim, Jae-Ho Bae, Dong-Wan Kim, Chi-Dug Kang, Sun-Hee Kim

**Affiliations:** 1 Department of Biochemistry, Pusan National University School of Medicine, Yangsan 626-870, South Korea; 2 Department of Microbiology, College of Natural Sciences, Chang Won National University, Chang Won 641-773, South Korea; 3 Medical Research Institute, Pusan National University School of Medicine, Yangsan 626-870, South Korea

## Abstract

**Background:**

The development of new modulator possessing high efficacy, low toxicity and high selectivity is a pivotal approach to overcome P-glycoprotein (P-gp) mediated multidrug resistance (MDR) in cancer treatment. In this study, we suggest a new molecular mechanism that TRAIL (tumor necrosis factor-related apoptosis-inducing ligand) down-regulates P-glycoprotein (P-gp) through inhibition of DNA-PKcs/Akt/GSK-3β pathway and activation of caspases and thereby sensitize MDR cells to MDR-related drugs.

**Results:**

MDR variants, CEM/VLB_10-2_, CEM/VLB_55-8 _and CEM/VLB_100 _cells, with gradually increased levels of P-gp derived from human lymphoblastic leukemia CEM cells, were gradually more susceptible to TRAIL-induced apoptosis and cytotoxicity than parental CEM cells. The P-gp level of MDR variants was positively correlated with the levels of DNA-PKcs, pAkt, pGSK-3β and c-Myc as well as DR5 and negatively correlated with the level of c-FLIPs. Hypersensitivity of CEM/VLB_100 _cells to TRAIL was accompanied by the activation of mitochondrial apoptotic pathway as well as the activation of initiator caspases. In addition, TRAIL-induced down-regulation of DNA-PKcs/Akt/GSK-3β pathway and c-FLIP and up-regulation of cell surface expression of death receptors were associated with the increased susceptibility to TRAIL of MDR cells. Moreover, TRAIL inhibited P-gp efflux function via caspase-3-dependent degradation of P-gp as well as DNA-PKcs and subsequently sensitized MDR cells to MDR-related drugs such as vinblastine and doxorubicin. We also found that suppression of DNA-PKcs by siRNA enhanced the susceptibility of MDR cells to vincristine as well as TRAIL via down-regulation of c-FLIP and P-gp expression and up-regulation of DR5.

**Conclusion:**

This study showed for the first time that the MDR variant of CEM cells was hypersensitive to TRAIL due to up-regulation of DR5 and concomitant down-regulation of c-FLIP, and degradation of P-gp and DNA-PKcs by activation of caspase-3 might be important determinants of TRAIL-induced sensitization of MDR cells to MDR-related drugs. Therefore, combination of TRAIL and chemotherapeutic drugs may be a good strategy for treatment of cancer with multidrug resistance.

## Background

Acquired resistance to chemotherapeutic agents remains a major obstacle for the effective treatment of many advanced and metastatic cancers. Several mechanisms are thought to be involved in the development of multidrug resistance (MDR), defined by simultaneous cross-resistance to a variety of anticancer drugs that differ in their chemical structures, modes of action, and molecular targets [1-3]. Emergence of MDR is often associated with over-expression of the *MDR1 *gene product, P-glycoprotein (P-gp) [4]. In certain cancers, such as chronic or acute myeloid leukemia and breast cancer, over-expression of *MDR1 *gene is a prognostic indicator for clinical outcome and correlates with a poor response to chemotherapy [5-8]. Therefore, inhibition of P-gp function or expression can reverse P-gp-mediated MDR and improve the efficacy of chemotherapy [9].

Previously, we have reported that an increased expression of DNA-dependent protein kinase (DNA-PK) participates in the development of MDR, and inhibition of DNA-PK leads to increase of drug sensitivity in MDR cells [10]. DNA-PK comprises a catalytic subunit (DNA-PK_cs_) with a DNA- binding Ku70 and Ku80 heterodimer acting as the regulatory element. It has been proposed that DNA-PK is a molecular sensor for DNA damage that enhances the signal via phosphorylation of many downstream targets [11]. Recently, it has been demonstrated that DNA-PKcs-catalyzed RNA Helicase A phosphorylation enhanced the transcription of the *MDR1 *gene through the CAAT-like element of the *MDR1 *gene promoter and thus DNA-PKcs played an important role in regulation of P-gp expression by *MDR1 *promoter activation [12].

The phosphoinositide 3-kinase (PI3K)/Akt pathway is also frequently implicated in tumorigenesis and chemotherapeutic resistance [13]. Recent studies have shown that there is a significant correlation between the phosphorylated, activated Akt and P-gp expression, and inhibition of the PI3K/Akt signaling pathway can reverse P-gp-mediated MDR [14-16]. Akt phosphorylation on Ser473 (S473) is required for activation of Akt, and a major Akt S473 kinase activity was found to be DNA-PK, a member of the PI3K-related kinase subfamily of protein kinases [17]. DNA-PKcs has been shown to colocalize with Akt and enhance Akt phosphorylation [18]. One of the downstream targets of pro-survival Akt is GSK-3β, which is inactivated by phosphorylation on Ser9 by Akt. The inactivation of GSK-3β through Akt-mediated phosphorylation leads to down-regulate its pro-apoptotic activity and inhibit the induction of cell death. Death receptor-induced extrinsic apoptotic signaling is also modulated by GSK-3β activity [19].

Recently, it has been shown that the extrinsic death receptor pathway represents a suitable target for cancer treatment [20]. Since TNF-related apoptosis inducing ligand (TRAIL) has been shown to induce apoptosis in various tumor cells, but only rarely in non-transformed cells, TRAIL is currently assessed in clinical trials [21]. The extrinsic apoptotic signaling cascade is a vital process initiated by activation of death receptors, DR4/DR5. Stimulation of these death receptors causes receptor trimerization, followed by recruitment of FADD (Fas associated with death domain protein) and caspase-8 (or caspase-10) to form the death-inducing signaling complex (DISC). DISC formation promotes autoactivation of caspase-8/10 and the subsequent activation of effector caspases, primarily caspase-3, -6 and -7, which implement the cell death program. Cellular FLICE inhibitory protein (c-FLIP) is expressed as long form (c-FLIP_L_) and short form (c-FLIP_S_) and inhibits caspase-8 binding to FADD and prevents DISC formation and apoptosis and splice forms [22]. Elevated Akt activity up-regulates c-FLIP and inhibits TRAIL-induced apoptosis in cancer cells [23].

It has been reported that MDR cells over-expressing P-gp are more susceptible to TRAIL than their drug-sensitive counterparts through various mechanisms such as a reduced expression of endogenous Akt [24] or enhancement of TRAIL binding to DR5 by P-gp [25]. However, the mechanism underlying the increased susceptibility of MDR cells to TRAIL mediated cell death was not understood well. Here, we demonstrated that TRAIL sensitized MDR cells to MDR-related drugs by inhibition of DNA-PKcs/Akt/GSK-3β pathway, activation of caspases and subsequent down-regulation of P-gp.

## Results

### The high susceptibility of MDR cells to TRAIL is associated with up-regulation of DR5 and down-regulation of c-FLIPs in the cells

**Figure 1:**
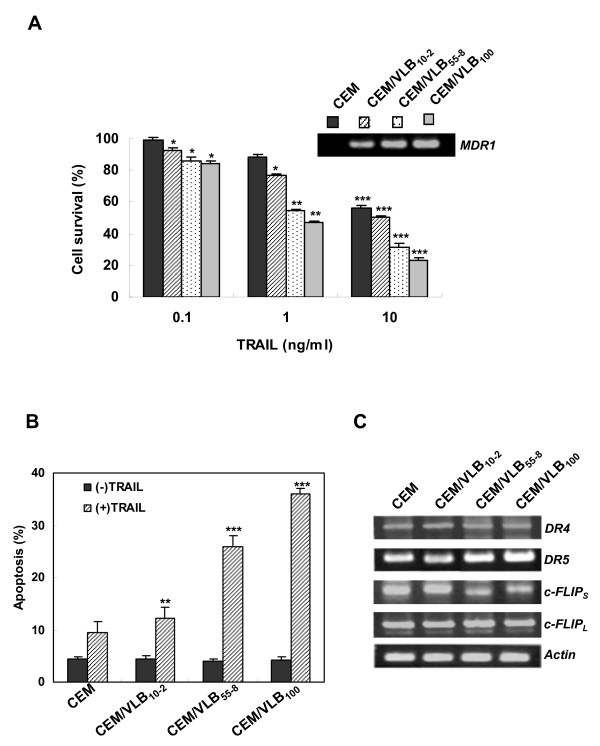
**High susceptibility of MDR cells to TRAIL and up-regulation of death receptor and down-regulation of c-FLIP in the cells**. **(A) **mRNA levels of mdr1 in CEM cells and its MDR variants, CEM/VLB_10-2_, CEM/VLB_55-8 _and CEM/VLB_100 _cells were determined by RT-PCR (*insert*). To determine the growth inhibitory effect of TRAIL, CEM cells and its multidrug-riesistant variants were treated with graded single doses of TRAIL, and the percentage of cell survival was determined after 5 days incubation using the MTT assay. **(B) **CEM cells and its MDR variants were treated with or without TRAIL (10 ng/ml) for 24 h. Thereafter, the percentage of apoptotic cells in each cell population was determined by Annexin V staining and flow cytometry. Each bar represents the mean ± S.D. of triplicate experiments. *p < 0.05, **p < 0.01, ***p < 0.005 versus TRAIL untreated control cells. **(C) **mRNA levels of DR4/DR5 and c-FLIP_L/S _in CEM cells and its MDR variants were determined by RT-PCR analysis. β-Actin (Actin) was used as a loading control.

Many studies have focused on overcoming MDR through modulation of P-gp function [4,26]. Since TRAIL as a targeted cancer therapy preferentially kills cancer versus normal cells [20], we compared the cytotoxic and apoptotic effects of TRAIL between CEM human lymphoblastic leukemic cell line and its multidrug resistant (MDR) variants, including CEM/VLB_10-2_, CEM/VLB_55-8_, and CEM/VLB_100 _isolated from parental CEM cells. In a model of CEM cells with gradually increasing expression of multidrug-resistance 1 (MDR1) gene, the higher expression level of MDR1 gene in MDR variants, the higher cytotoxicity to TRAIL as shown in MTT assay (Figure 1A) and flow cytometric analysis of apoptosis (Figure 1B), demonstrating that the MDR cells were more susceptible to TRAIL-induced cell death than TRAIL-insensitive parental cells. Since TRAIL acts through the TRAIL receptors (DR5 and DR4) to induce apoptosis [20], we compared the mRNA levels of DR5 and DR4 between CEM and its MDR variants (Figure 1C). The level of DR5 but not DR4 in MDR variants was significantly increased as compared with that in CEM cells. This result suggests that an increased level of DR5 in MDR cells plays an important role in the sensitization of the cells to TRAIL. To understand further the factors that contribute to the sensitization of MDR cells to TRAIL, we determined the expression of c-FLIP_L/S_, an important downstream regulator of death receptor-mediated apoptosis [27], in MDR variants. We found that the mRNA level of c-FLIP_S_, but not c-FLIP_L_, was significantly decreased in MDR variants as compared to that in CEM cells. Therefore, our data indicated that TRAIL susceptibility of MDR cells was closely associated with up-regulation of DR5 and down-regulation of c-FLIPs in the cells.

### TRAIL-induced apoptosis in MDR cells is mediated through caspase activation and mitochondrial pathway

**Figure 2:**
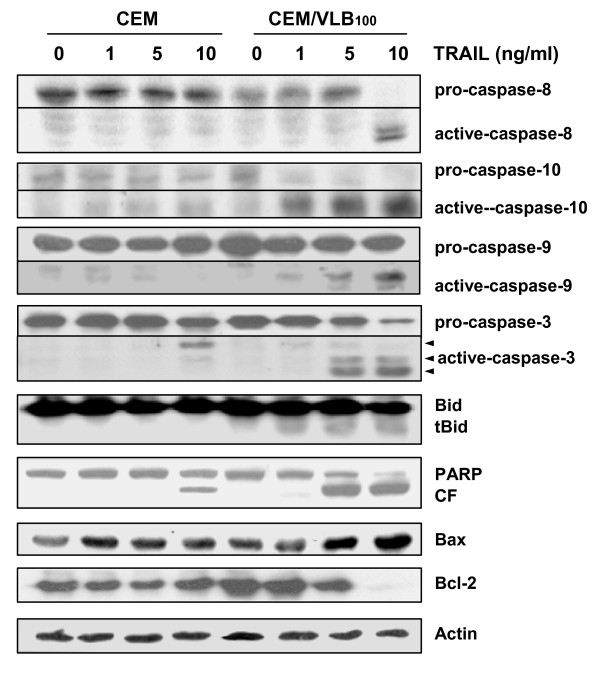
**Sensitization of MDR cells to TRAIL is mediated through caspase-dependent mitochondrial apoptotic pathway**. The cell lysates obtained from CEM or CEM/VLB_100 _cells after exposure to graded doses of TRAIL (1~10 ng/ml) for 24 h were subjected to western blot analysis to monitor levels of caspase-8, -10, -9 and -3, Bid, truncated Bid (tBid), Bax and Bcl-2. The levels of PARP and its cleavage fragment (CF) in TRAIL-treated cells were also determined.

We determined if the increased susceptibility to TRAIL-induced apoptosis of CEM/VLB_100 _cells was accompanied by activation of caspases and the mitochondrial apoptotic pathway (Figure 2). Consistent with the more sensitive response of CEM/VLB_100 _cells to TRAIL-induced cell death, cleavage and activation of procaspase-8 and -10, which are initiator caspases linked to receptor mediated apoptotic pathway, occurred remarkably in CEM/VLB_100 _cells as compared to CEM cells. In case of caspase-9 that has been linked to the mitochondrial death pathway, treatment of CEM/VLB_100 _cells with TRAIL resulted in significant induction of proteolytic processing of caspase-9 to its active form compared to CEM cells, which did not show proteolytic cleavage of procaspase-9. Finally, procaspase-3, an executioner caspase, and PARP, a hallmark of caspase activation, were cleaved profoundly in CEM/VLB_100 _cells compared with CEM cells.

Meanwhile, we investigated whether the modulation of Bcl-2 family proteins is involved in TRAIL-induced apoptosis of MDR cells. Down-regulation of Bcl-2 and up-regulation of Bax apparently occurred after treatment with TRAIL in CEM/VLB_100 _cells but not in CEM cells and these results were followed by TRAIL-induced truncation of Bid in CEM/VLB_100 _cells but not in CEM cells, consistent with activation status of procaspase-9 in CEM/VLB_100 _cells and CEM cells after treatment with TRAIL. Therefore, these data indicated that TRAIL-induces apoptosis occurred in the MDR cells through caspase-dependent mitochondrial pathway as well as receptor-mediated apoptotic pathway.

### The increased expression of DNA-PKcs is associated with up-regulation of P-gp and c-Myc expression via Akt/GSK-3β pathway in MDR cells

**Figure 3:**
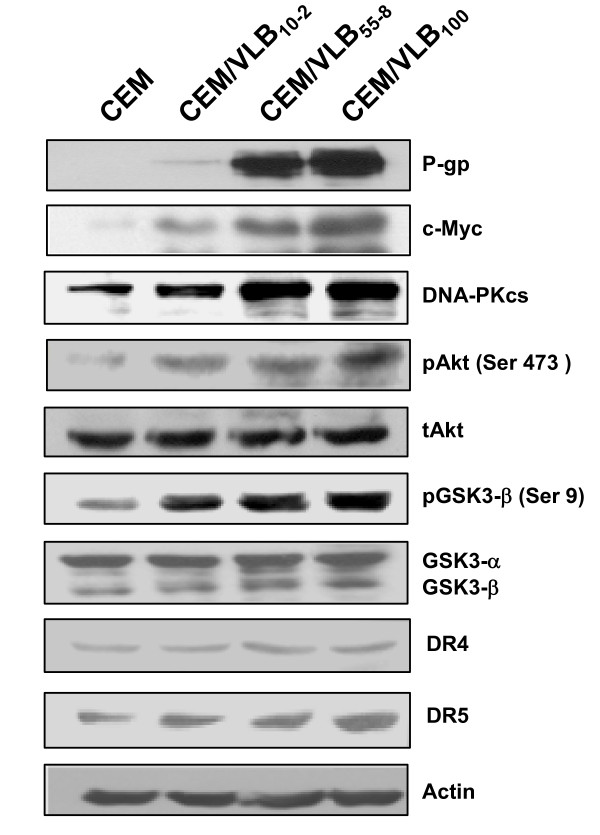
**Expression of p-gp in MDR cells is associated with the enhanced expression of c-Myc and DR5 and activation of DNA-PKcs/Akt/GSK-3β pathway**. The protein levels of P-gp, c-Myc, DNA-PKcs, pAkt, GSK-3β, phosphorylated Akt (pAkt) at Ser 473, total Akt (tAkt), phosphorylated GSK-3β (pSK3β) at Ser9, total GSK-3α/β, DR4 and DR5 were determined by western analysis. Actin was used as a loading control.

It has been reported DNA-PKcs regulates c-Myc stability via phosphorylation of Akt on Ser473, which in turn inactivates GSK-3β by the phosphorylation of GSK-3b on Ser9, resulting in stabilization of c-Myc [28]. The c-Myc is known to be involved in regulating expression of P-gp, the product of *MDR1 *gene [29,30] and renders cells sensitive to TRAIL-induced apoptosis [31]. Since the increased activity of DNA-PK participates in the development of MDR phenotype [10], we determined the relationships among P-gp, c-Myc and DNA-PKcs/Akt/GSK-3β molecules in MDR variants (Figure 3). We found that the gradually increased level of P-gp in CEM/VLB_10-2_, CEM/VLB_55-8 _and CEM/VLB_100 _cells was well correlated with the level of c-Myc in the each MDR variant. Therefore, we examined whether the basal levels of DNA-PKcs, phosphorylated Akt (pAkt) and phosphorylated GSK-3β (pGSK-3β) in MDR variants were compared with those in parental CEM cells. The basal level of DNA-PKcs was gradually higher in CEM/VLB_10-2_, CEM/VLB_55-8 _and CEM/VLB_100 _cells than in CEM cells, which was followed by gradual increase in pAkt and pGSK-3β levels without change in total Akt (tAkt) and GSK-3α/β levels. We also observed that the basal level of DR5 but not DR4 in MDR variants was significantly increased as compared with those in CEM cells. These results suggested that the increased expression of DNA-PKcs in MDR cells might lead to up-regulation of P-gp and c-Myc expression via phosphorylation of Akt and GSK-3β proteins.

### TRAIL-induced down-regulation of DNA-PKcs/Akt/GSK-3β pathway and c-FLIP and up-regulation of DR4/DR5 cell surface expression are associated with the susceptibility to TRAIL of MDR cells

**Figure 4:**
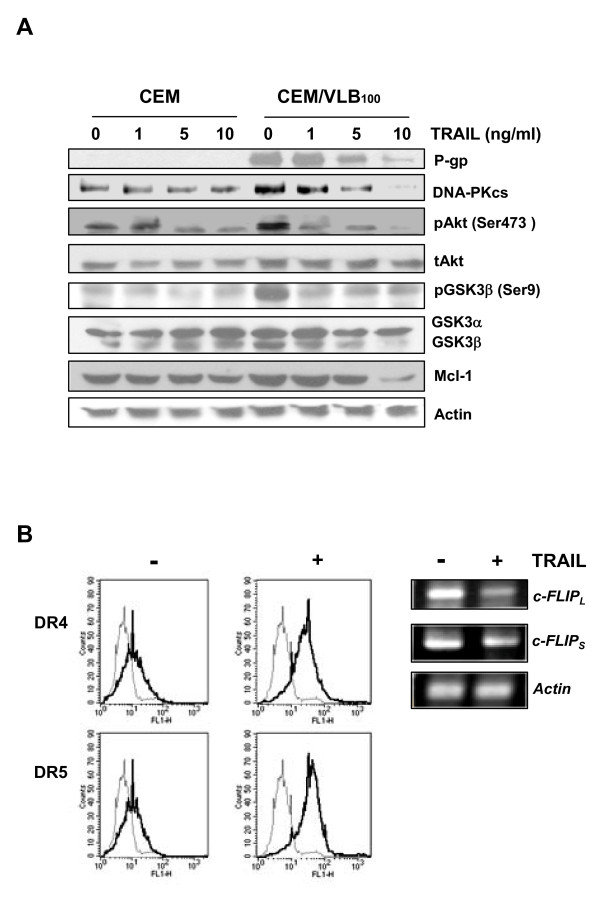
**TRAIL inhibited P-gp expression and DNA-PKcs/Akt/GSK-3β signaling pathway and up-regulation of death receptors and down-regulation of c-FLIP in MDR cells**. **(A) **Cell lysates obtained from CEM or CEM/VLB_100 _cells treated with indicated dose of TRAIL for 24 h were subjected to western blot analysis to monitor levels of P-glycoprotein (P-gp), DNA-PKcs, pAkt (Ser473), tAkt, pGSK3β (Ser 9), GSK-3α and -3β, Mcl-1. **(B) **CEM/VLB_100 _cells treated with TRAIL (10 ng/ml) for 24 h were incubated on ice in the presence of DR4- and DR5-specific antibodies (1:500), and subsequently labeled with FITC-conjugated secondary antibody (1:1000). The fluorescence intensity was analyzed with flow cytometry. The thin line indicates that the cells only incubated with Goat IgG2a that was used as a control isotype antibody; the thick line indicates the specific labeling (*left*). The cells treated with or without TRAIL, and changed mRNA level of c-FLIP_L/S _was determined by RT-PCR analysis (*right*).

Since our data showed that the level of DR5 was well correlated with the activity of the DNA-PKcs/Akt/GSK-3β pathway in MDR cells, we determined whether the levels of DNA-PKcs, Akt as a downstream target of DNA-PKcs, and GSK-3b, a downstream target of the Akt pathway, are modulated after treatment of CEM and CEM/VLB_100 _cells with TRAIL (Figure 4A). When both CEM and CEM/VLB_100 _cells were treated with TRAIL, the levels of DNA-PKcs, pAkt and pGSK-3b as well as P-gp were significantly decreased in CEM/VLB_100 _cells as compared with those of CEM cells in a dose-dependent manner. The decrease of pAkt was not followed by decrease of tAkt, but the decrease of pGSK-3b seemed to be associated with decrease of total GSK-3b. These results were followed by down-regulation of Mcl-1, one of the anti-apoptotic Bcl-2 family, that plays a key role in acquired TRAIL resistance [32]. These results suggest that down-regulation of both P-gp and DNA-PKcs/Akt/GSK-3β pathway by TRAIL might be played an important role in death receptor-mediating TRAIL-induced apoptosis in the P-gp over-expressing MDR cells with high expression of c-Myc. Meanwhile, treatment of CEM/VLB_100 _cells with TRAIL led to a significant suppression of mRNA level of c-FLIP_L _and c-FLIP_S _and an increase in surface expression of DR4 and DR5 (Figure 4B). These results suggest that an increase in cell surface expression of DR4/DR5 and down-regulation of c-FLIP and DNA-PKcs/Akt/GSK-3β pathway by TRAIL play an important role in death receptor-mediating TRAIL-induced apoptosis in the MDR cells.

### TRAIL inhibited P-gp efflux function via degradation of P-gp and potentiated the cytotoxicity of MDR-related drug in MDR cells

**Figure 5:**
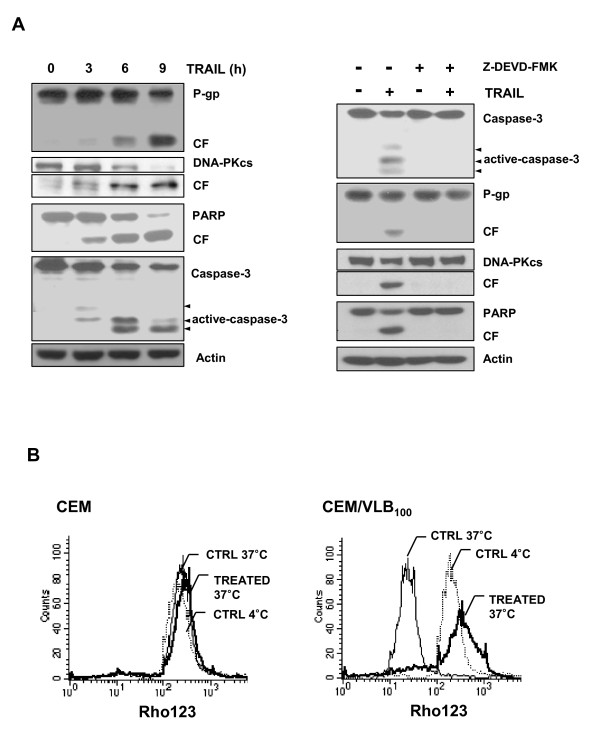
**TRAIL inhibited P-gp efflux function in MDR cells by P-gp cleavage via caspase-3 activation**. **(A) **The cell lysates obtained from CEM/VLB_100 _cells treated with or without TRAIL (10 ng/ml) for 3 ~ 9 h (*left*) and the cell lysates of the MDR cells treated with TRAIL (5 ng/ml) for 6 h or pretreated with 50 μM Z-DEVD-FMK, a specific caspase-3 inhibitor, for 3 h and then with TRAIL for 6 h (*right*) were subjected to western blot analysis to monitor levels of P-gp, DNA-PKcs, caspase-3 and PARP and their its cleavage fragment (CF). **(B) **flow cytometric assay of P-gp efflux activity in TRAIL-treated MDR cells was based on extrusion of the fluorescent P-gp substrate, rhodamine123 (Rho123). The efflux activity of P-gp is highly temperature sensitive because functions optimally 37°C but is inactive at 4°C. Cell suspension from CEM and CEM/VLB_100 _cells treated with or without 10 ng/ml TRAIL for 6 h was incubated with Rho 123 and further incubated at 37°C for 3 h (TREATED 37°C as TRAIL-treated cells or CTRL37°C as TRAIL-untreated control) to allow P-gp-mediated drug efflux or on ice as control (CTRL 4°C).

Since P-gp cleavage is dependent on caspase-3 activity following treatment with apoptosis inducers [33], we hypothesized that TRAIL-induced caspase-3 activation in CEM/VLB_100 _cells may lead to the degradation of P-gp. When the MDR cells were treated with TRAIL, the cleavage of P-gp was accompanied with induction of proteolytic cleavage of procaspase-3 to the active forms. In addition, the cleavage of DNA-PKcs and PARP, well known endogenous substrates of caspase-3 [34,35], was observed in TRAIL-treated CEM/VLB_100 _cells (Figure 5A, *left*). These results were followed by prevention of cleavage of P-gp as well as DNA-PKcs and PARP by pretreatment with Z-DEVD-FMK, a caspase-3-specific inhibitor (Figure 5A, *right*). Therefore, we determined whether TRAIL inhibited P-gp efflux function of the MDR cells, using a flow cytometric functional efflux assay based on the extrusion of the fluorescent substrate, rhodamine 123 (Figure 5B). P-gp functions optimally near 37°C, but it is inactive at 4°C. When P-gp expressing CEM/VLB_100 _cells are preloaded with rhodamine 123 and incubated at 4°C, they retain the dye and consequently have high fluorescence levels. But, CEM/VLB_100 _cells incubated at 37°C more readily efflux the dye and show reduced fluorescence. Treatment with TRAIL significantly reduced rhodamine123 efflux and thereby increased the accumulation of rhodamine123 in CEM/VLB_100 _cells. When we performed this functional assay also on CEM cells, which does not express P-gp, there were no differences in the fluorescence intensity between the three experimental conditions. Therefore, this result suggests that the inhibitory effect of TRAIL on P-gp efflux function might be due to caspase-3-dependent P-gp cleavage.

**Figure 6:**
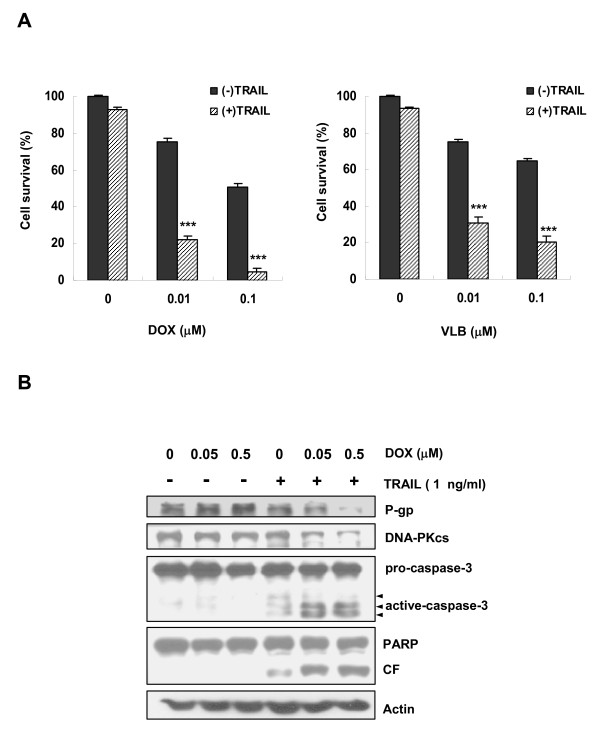
**TRAIL enhanced the cytotoxicity of MDR-related drug in MDR cells by the down-regulation of DNA-PKcs and P-gp via caspase-3 activation**. **(A) **CEM/VLB_100 _cells were treated with graded single doses of doxorubicin (DOX) or vinblastine (VLB) after pretreatment of low dose of TRAIL (1 ng/ml) for 6 h. The percentage of cell survival after combined treatment of TRAIL with MDR-related drug was determined after 5 days incubation using the MTT assay. Each bar represents the mean ± S.D. of triplicate experiments. ***p < 0.005 versus TRAIL alone treated cells at the same dose point. **(B) **The cell lysates obtained from CEM/VLB_100 _cells co-treated with TRAIL (1 ng/ml) and indicated dose of DOX were subjected to western blot analysis to monitor levels of P-gp, DNA-PKcs, caspase-3, PARP, and actin as a loading control.

We next examined whether the cytotoxicity of the MDR-related drugs such vinblastine (VLB) and doxorubicin (DOX) could be enhanced by pretreatment of TRAIL (Figure 6A). We found that the cytotoxicity of VLB and DOX was significantly enhanced in CEM/VLB_100 _cells by pre-treatment with low dose of TRAIL (1 ng/ml). These results suggest that inactivation of P-gp by TRAIL may be a cause of sensitization of CEM/VLB_100 _cells to MDR-related drugs, and thus the use of TRAIL in combination with MDR-related drug for growth inhibition in MDR cells might be overcome the drug resistance of the MDR cells. In addition, to reveal the synergistic cytotoxic mechanisms of the combined treatment of TRAIL with MDR-related drug against MDR cells, we determined the change of activity of caspase-3 and expression of P-gp, DNA-PKcs and PARP in CEM/VLB_100 _cells after the combined treatment with MDR-related drug and TRAIL (Figure 6B). As expected, the combined treatment with DOX and TRAIL was more effective than either treatment alone to down-regulate P-gp and DNA-PKcs and to increase subsequent PARP cleavage via caspase-3 activation in the MDR cells. Therefore, these results suggest that TRAIL might be effective for overcoming the MDR phenotype of cancer cells by combination with MDR-related drug.

### Suppression of DNA-PKcs by siRNA enhanced the susceptibility of MDR cells to MDR-related drug as well as TRAIL via up-regulation of DR5 and down-regulation of c-FLIP and P-gp expression

**Figure 7:**
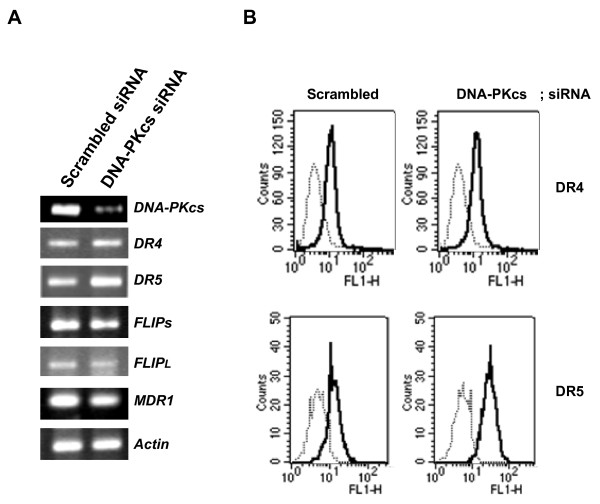
**Supprerssion of DNA-PKcs up-regulated surface expression of DR5 and down-regulated the expression of c-FLIPs**. **(A) **CEM/VLB_100 _cells were transfected with a siRNA against DNA-PKcs or scrambled siRNA as a control. After 48 h, the total RNA extracted from transfectant of CEM/VLB_100 _cells performed RT-PCR analysis to monitor the mRNA levels of DNA-PKcs, DR4/5, c-FLIP_L/S _, MDR1, and actin as a loading control. **(B) **The transfectant incubated with an anti-DR4 or -DR5 (1:500), and subsequently labeled with FITC-conjugated secondary antibodies (1:1000) to determine the surface expression of DR4 and DR5. Goat IgG2a was also used as control isotype antibody.

**Figure 8:**
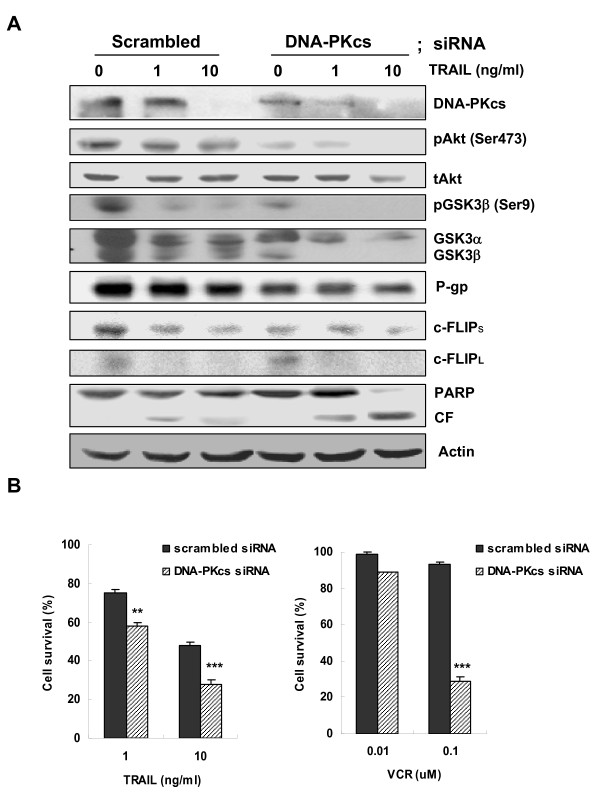
**Potentiated suppression of DNA-PKcs expression by TRAIL leads to a severe inhibition of both Akt/GSK-3β phosphorylation and P-gp and c-FLIPs expression and enhances the cytotoxicity of TRAIL and of MDR-related drug in MDR cells**. **(A) **CEM/VLB_100 _cells were transfected with a siRNA against DNA-PKcs or scrambled siRNA as a control. After 48 h, the transfectant was treated with or without TRAIL (1- and 10 ng/ml for 24 h), and the changed level of DNA-PKcs, pAkt (Ser473), tAkt, pGSK3β (Ser9), GSK-3α/β, P-gp, PARP, c-FLIP_L/S _and actin was performed by western blot analysis. **(B) **Each transfectant was treated with indicated doses of TRAIL or vincristine (VCR). After 5 days, the percentage of growth inhibition was determined incubation using the MTT assay. Data represent means ± S.D. of triplicate experiments. **p < 0.001, ***p < 0.005 versus cells transfected with scrambled siRNA at the same dose point.

To determine the role of DNA-PKcs on the expression of c-FLIP_L/S _and DR4/DR5, which are major determinants of responsiveness to TRAIL, and *MDR1*, we silenced DNA-PKcs in CEM/VLB_100 _cells using small interfering RNA (siRNA) and determined the changed mRNA levels of the genes using RT-PCR analysis (Figure 7A). The apparent increase in the mRNA level of DR5 was observed in the MDR cells after transfection with DNA-PKcs siRNA compared with scrambled siRNA, and DR4 also increased slightly. Conversely, the mRNA level of c-FLIP_S _but not c-FLIP_L _in CEM/VLB_100 _cells was significantly reduced after transfection with DNA-PKcs siRNA. We also found that siRNA-mediated silencing of DNA-PKcs significantly down-regulated the expression of *MDR1 *gene in the MDR cells. Moreover, the increased transcription of DR5 gene was followed by increased cell surface expression of DR5 in CEM/VLB_100 _cells after transfection with DNA-PKcs siRNA (Figure 7B). These results suggest that the down-regulated DNA-PKcs after treatment with TRAIL may play important roles in the regulation of death receptors and c-FLIP as well as *MDR1 *gene expression, and the inhibition of DNA-PKcs in MDR cells may enhance the susceptibility to TRAIL as well as MDR drugs via up-regulation of DR5 and down-regulation of c-FLIP and P-gp expression, respectively. In addition, we demonstrated that the inhibition of DNA-PKcs by transfection with DNA-PKcs siRNA caused the reduction of pAkt, pGSK-3β and P-gp levels in CEM/VLB_100 _cells (Figure 8A). The reduction of pAkt level was not followed by the reduction of total Akt, while the reduction of pGSK-3β level was associated with the reduction of total GSK-3. In addition, suppression of DNA-PKcs led to the decrease in P-gp and c-FLIPs and a concurrent increase in cleaved PARP, which was accelerated by TRAIL. These results suggest that suppression of DNA-PKcs would lead to increased susceptibility to TRAIL-induced cytotoxicity in MDR cells. Therefore, we next examined whether siRNA-mediated suppression of DNA-PKcs affects the susceptibility of CEM/VLB_100 _cells to TRAIL-induced cytotoxicity. After transfection with DNA-PKcs siRNA or scrambled siRNA, the transfected cells were treated with indicated doses of TRAIL for 5 days. The susceptibility to TRAIL-induced cytotoxicity of CEM/VLB_100 _cells was significantly increased after transfection with DNA-PKcs siRNA (Figure 8B, *left*). Furthermore, we also confirmed whether siRNA-mediated suppression of DNA-PKcs affects the susceptibility of CEM/VLB_100 _cells to vincristine (VCR) (Figure 8B, *right*). These results suggest that targeting of DNA-PKcs could enhance the susceptibility of MDR-related drug as well as TRAIL on P-gp over-expressing MDR cells with high expression of DNA-PKcs.

## Discussion

Although targeted drugs are being developed or used in some leukemia, chemotherapeutic drugs are still useful for the treatment of leukemia. However, acquired resistance against MDR-related drugs is a serious problem in the management of leukemic patients. Altered expression of various kinds of protein and enzymes could be seen in MDR-type cancer cells [2,9]. In the present study, we suggest a new molecular mechanism that TRAIL down-regulates P-gp through inhibition of DNA-PKcs/Akt/GSK-3β pathway and activation of caspases and thereby sensitize MDR cells to MDR-related drugs.

TRAIL is emerging as most promising agent for cancer therapy, because it induces apoptosis in a variety of cancers and transformed cells without any toxicity to normal cells [20]. But, it has been reported that a majority of human leukemic cells such as CEM, K562 and Molt-4 cells are relatively resistant to TRAIL-induced apoptosis [36,37]. In our study, interestingly, MDR variants derived from human lymphoblastic leukemia CEM cells showed a hypersensitive response to TRAIL compared with parental CEM cells. MDR variants, CEM/VLB_10-2_, CEM/VLB_55-8 _and CEM/VLB_100 _cells with gradually increased levels of P-gp were gradually more susceptible to TRAIL-induced apoptosis and cytotoxicity than CEM cells. This result was supported by the findings that the expression of DR5 was gradually up-regulated in the CEM/VLB_10-2_, CEM/VLB_55-8 _and CEM/VLB_100 _cells, and conversely, the expression of c-FLIPs was gradually down-regulated in the MDR variants as compared with those of CEM cells. Therefore, modulation of TRAIL receptor pathway including up-regulation of DR5 and down-regulation of c-FLIPs might contribute to TRAIL sensitization of MDR cells. It has been reported that TRAIL responsiveness correlates with a reduced expression of endogenous Akt in MDR-U2OS human osteosarcoma cell line [24], and P-gp enhances TRAIL-triggered apoptosis by interacting with the death receptor DR5 in the *MDR1*-transfected MCF-7 breast cancer cell line [25]. Therefore, it could be suggested that a marked sensitivity to TRAIL of MDR cells might be mediated by complex mechanisms, not a single mechanism. In the present study, hypersensitivity to TRAIL of CEM/VLB_100 _cells, MDR variant of CEM cells, was accompanied by the activation of the mitochondrial apoptotic pathway by the cleavage of bid as well as the activation of caspase-8 and -10, which are apoptotic characteristics of the type II cells and caspase-3 and -9 [38]. We also observed an increase in cell surface expression of DR4/DR5 and down-regulation of c-FLIP by TRAIL in MDR-variant of CEM cells. These results suggest that there might be a positive feedback regulation in TRAIL receptor signaling leading to intensification of sensitivity to TRAIL in MDR-variant of CEM cells.

Oncogene c-Myc is known to act as an important regulator for TRAIL sensitivity in cancer cells. It has been shown that c-Myc induces and represses the transcription of DR5 [39] and c-FLIP [40], respectively, therefore enhancing the sensitivity of cancer cells to TRAIL-induced apoptosis. Recently, it has been reported that abnormal overexpression of DNA-PKcs may contribute to cell proliferation and even oncogenic transformation by stabilizing the c-Myc oncoprotein via at least the Akt/GSK3 pathway [28]. Previously, we have demonstrated that the increased expression of DNA-PKcs is associated with the development of drug resistance in MDR variants of CEM cells [10]. In addition, the c-Myc is known to be involved in regulating expression of P-gp, the product of *MDR1 *gene [29,30]. It has been reported that elevated P-gp expression in MDR cells is accompanied by increased level of pAkt [41]. Once phosphorylated, activated Akt inactivate GSK-3β through phosphorylation at Ser9, resulting in stabilization and activation of β-catenin that enhanced P-gp expression [42]. In the present study, the gradually increased level of P-gp, was well correlated with the gradually increased levels of c-Myc, DNA-PKcs, pAkt and pGSK-3β in MDR variants, CEM/VLB_10-2_, CEM/VLB_55-8 _and CEM/VLB_100 _cells, suggesting that the molecular changes are not dependent on the each subline type, but implicate the causal relationships between the molecules, which have been changed during the process of MDR acquisition. And the increased level of DR5 and decreased level of c-FLIPs in the MDR-variants of CEM cells also might be associated with the up-regulated c-Myc since it has been reported that c-Myc up-regulated the DR5 receptor and down-regulated c-FLIP [39,40]. We also found that the expression of up-regulated molecules in CEM/VLB_100 _cells including P-gp, DNA-PKcs, pAkt and pGSK-3β were suppressed after treatment with TRAIL. Akt and GSK-3β are signaling molecules downstream to DNA-PKcs. We showed that the phosphorylated form of Akt and GSK-3β would be decreased in TRAIL-treated CEM/VLB_100 _cells since DNA-PKcs was down-regulated by TRAIL treatment. Therefore, our data indicated that TRAIL caused the down-regulation of P-gp in MDR cells by the inactivation of DNA-PKcs/Akt/GSK-3b pathway. Since these molecules are related with drug-resistance, down-regulation of P-gp, DNA-PKcs, pAkt, and pGSK-3β after treatment with TRAIL might lead to the hypersensitivity to MDR-related drugs of MDR-variant of CEM cells. Indeed, inhibition of Akt enhances susceptibility to TRAIL by up-regulation of death receptors [43] and down-regulation of c-FLIP [44] and down-regulates P-gp expression in multidrug-resistant human T-acute leukemia [14].

Our study also showed that anti-apoptotic Bcl-2 and Mcl-1 proteins were over-expressed in CEM/VBL_100 _cells and the levels of these proteins and Bax were significantly decreased and increased by Bcl-2 and Mcl-1, the antiapoptotic Bcl-2 family proteins, were over-expressed in CEM/VBL_100 _cells in comparison with CEM cells, and the levels of these anti-apoptotic proteins and Bax were significantly decreased and increased by treatment of TRAIL in CEM/VBL_100 _cells, respectively, suggesting that TRAIL-induced apoptosis of MDR cells was mediated through mitochondria-dependent pathway as well as caspase activation. Bcl-2 and Mcl-1 are often highly expressed in chemotherapy-resistant cancers and prevents apoptosis by inactivating pro-apoptotic Bax and Bak [45,46]. The increased expression of Bcl-2 or Bcl-xL was the common feature of P-gp-related drug-resistant human leukemic ce1l lines [47]. Over-expression of Mcl-1 decreased sensitivity of leukemia cells to cytotoxic chemotherapeutic agents [45] and specific down-regulation of Mcl-1 via RNA interference sensitized multidrug-resistant leukemia cells towards chemotherapy and induced apoptosis [48]. Therefore, the reduction of Bcl-2 and Mcl-1 after exposure to TRAIL may be in part a cause of TRAIL-induced sensitization of CEM/VLB_100 _cells to MDR-related drugs.

Moreover, our data showed the cleavage of P-gp and DNA-PKcs by treatment with TRAIL. Recently, it has been shown that the cleavage of P-gp (170 kDa) is dependent on caspase-3 during apoptotic cell death induced by LY294002, H_2_O_2_, and Z-LEHD-FMK in MDR variant of CEM cells [33]. DNA-PKcs is also a substrate of caspase-3 [35]. Here, we demonstrated that the degradation of P-gp and DNA-PKcs during treatment of CEM/VLB_100 _cells with TRAIL was a caspase-3 dependent manner. This result was followed by the significant reduction of rhodamine123 efflux and the increased sensitivity to MDR-related drugs such as VLB and DOX after exposure to TRAIL in CEM/VLB_100 _cells, suggesting that the degradation of P-gp as well as the down-regulation of Bcl-2 and Mcl-1 could be involved in sensitization of MDR cells to MDR-related drug after treatment with TRAIL. Since the expression of DNA-PKcs regulates *MDR1 *gene [12] and cellular c-Myc protein levels [28], which can affect TRAIL sensitivity in cancer cells as described above, we showed that knockdown of DNA-PKcs with specific siRNA led to the increased expression of DR5 and the decreased expression of c-FLIP and caused the reduction of pAkt, pGSK-3β and P-gp levels in CEM/VBL_100 _cells. These results resembled the effects of TRAIL on the CEM/VBL_100 _cells. Therefore, DNA-PKcs may play an important role on TRAIL sensitivity in MDR-variant of CEM cells.

## Conclusions

In conclusion, this study showed for the first time that the MDR-variants of CEM cells with an increased P-gp and c-Myc were hypersensitive to TRAIL and that the degradation of both P-gp and DNA-PKcs after exposure to TRAIL might be an important determinant of susceptibility to TRAIL-induced apoptosis in MDR cells. In addition, the treatment with TRAIL caused severe down-regulation of the up-regulated P-gp and DNA-PKcs in MDR cells and consequently sensitized the MDR cells to MDR-related drugs. Therefore, combination of TRAIL and chemotherapeutic drugs may be a good strategy for treatment of cancer with multidrug resistance, and DNA-PKcs/Akt/GSK-3β signaling pathway may be an important target to overcome multidrug resistance.

## Methods

### Cell culture and Reagents

Human lymphoblastic leukemia CCRF-CEM (CEM) line and its the multidrug-resistant sublines, CEM/VLB_10-2_, CEM/VLB_55-8_, and CEM/VLB_100 _[10], were cultured in RPMI 1640 medium supplemented with 10% fetal bovine serum (FBS, GIBCO BRL, Life Technologies, Inc.). The recombinant human soluble TRAIL was purchased from R&D System (Minneapolis, MN). Vinblastine, vincristine, doxorubicin and Rho123 were obtained from Sigma-Aldrich (St. Louis, MO).

### Cell Proliferation Assay

Cell proliferation was measured either by counting viable cells by using the 3-(4, 5-dimethylthiazol-2-yl)-2,5-diphenyltetrazolium bromide (MTT; Sigma Chemical Company, St. Louis, MO) colorimetric dye-reduction method. Exponentially growing cells (5 × 10^3 ^cells/well) were plated in 96 well and incubated in growth medium treated with indicated condition of TRAIL and/or drug at 37°C. After 5 days, the medium was aspirated using centrifugation and MTT-formazan crystals solubilized in 100 μl DMSO. The optical density of each sample at 570 nm was measured using ELISA reader. The optical density of the media was proportional to the number of viable cells. Inhibition of proliferation was evaluated as a percentage of control growth (no drug in the sample). All experiments were repeated at least two experiments in triplicate.

### Flow cytometric analysis of TRAIL receptors

CEM/VLB_100 _cells (2 × 10^6 ^cells) from the culture media were spun down at 500 × g, washed with phosphate-buffered saline (PBS) and resuspended in 500 μl PBS. The cells were then incubated with 5 μl of goat IgG2a, anti-DR4 or anti-DR5 polyclonal goat antibody (1:100, R&D, Minneapolis, MN) for 1 h. After washing with PBS, FITC-conjugated rabbit anti-goat polyclonal antibody (1:200, Sigma-Aldrich Co., St. Louis, MO) was added to the cell suspension and incubated for 1 h on ice. After rinsing with PBS, the samples were analyzed with a FACSort flow cytometer (Becton Dickinson, San Jose, CA). The data were analyzed using the CellQuest program.

### RT-PCR analysis

Total cellular RNA was isolated using RNeasy Mini Kit (Qiagen, Hilden, Germany) according to the manufacturer's protocol and the levels of RNA transcripts were assessed with The Titan One Tube RT-PCR System (MJ research Inc, NV, USA). One mg of total cellular RNA was reverse transcribed using Maloney murine leukemia virus reverse transcriptase (Invitrogen, Paisley, UK) with each dNTP and 1 μg oligo dT. Amplification of 1 μl of these cDNA by PCR was performed using the following gene-specific primers: DR4 (forward), 5'-CTGAGCAACGCAGACTCGCTGTCCAC-3' and (reverse), 5'-AAGGACACGGCAGAGCCT GTGCCAT-3'; DR5 (forward), 5'-CTGAAAGGCATCTGCTCAGGTG-3' and (reverse), 5'-CA GAGTCTGCATTACCTTCTA G-3'; FLIP_L _(forward), 5'-TTCCAGGCTTT CGGTTTCTT-3' and (reverse), 5'-GTCCGAAACAAGGTGAGGGT-3'; FLIP_S _(forward), 5'-ACCCTCACCTTG TTTCGGAC-3' and (reverse), 5'-CTTTTGGATTGCTGCTTGGA-3'; β-actin (forward), 5'-CAGAGCAAGAGAGGCATCCT-3' and (reverse), 5'-TTGAAGGTCTC AAACATGAT-3.'

The resulting total cDNA was used in PCR performed in total volume of 20 μl using Taq polymerase (Solgent Co., Korea) at 94°C for denaturation for 60 sec, 60°C for annealing for 60 sec, and 72°C for amplification for 90 sec for 30 cycles, followed by a final extension at 72°C for 12 min. The amplified fragments were separated on 1.5% agarose gel and visualized with ethidium bromide staining.

### Western blot analysis

Protein samples were separated by SDS-PAGE and blotted to nitrocellulose membrane (Hybond-ECL, GE Healthcare). The membrane was incubated with antibody as specified, followed by secondary antibody conjugated with horseradish peroxidase. Specific antigen-antibody complexes were detected by enhanced chemiluminescence (PerkinElmer, Life science). Western blot analysis was performed with the following antibodies: anti-Bax, anti-caspase-3, anti-PARP, anti-Bcl-2 (Santa Cruz Biotechnology, CA), anti-Akt, anti-phospho-Akt (Ser 473), anti-caspase-8, anti-caspase-9 (Cell signal, Danvers, MA), anti-DNA-PKcs (Thermo Fisher Scientific, CA), anti-DR5, anti-caspase-10 (Calbiochem, Germany), anti-pGSK-3β (Ser8), anti-GSK-3β, anti-GSK-3α, anti-c-Myc (Epitomics, CA), anti-DR4 (R&D Systems, MN) and anti β-actin (Sigma-Aldrich) antibodies, Secondary antibodies were obtained from GE Healthcare.

### Preparation of siRNA Transfection

The siRNA used for targeted silencing of DNA-PKcs were (5'-CAGUCUUAGUCCGGAUCAUdTdT-3'). CEM/VLB_100 _cells were transfected with 0.1 uM siRNA for 48 h by oligofectamine according to the manufacture's protocol (InVitrogen, Carlsbad, CA). In brief, CEM/VLB_100 _(2 × 10^5 ^cells/well) were seeded of 6-well plates and added to the siRNA/oligofectamine complex. Cells were incubated for 4 h at 37°C in serum free RPMI medium and then FBS was added. After 48 h, the cells were treated with TRAIL for another 24 h and collected for western blot analysis to determine the levels of DNA-PKcs and other indicated proteins.

### Apoptosis assay

Cells (2 × 10^5 ^cells/ml) were treated with or without TRAIL and/or indicated drug for 24 h and the cells were centrifuged and resuspended in 500 μl of the staining solution containing Annexin V fluorescein (FITC Apoptosis detection kit; BD ParMingen San Diego, CA) and propidium iodide in PBS. After incubation at room temperature for 15 min, cells were analyzed by flow cytometry. Annexin V binds to those cells that express phosphatidyl serine on the outer layer of the cell membrane, and propidium iodide stains the cellular DNA of those cells that have a compromised cell membrane. This allows for the discrimination of live cells (unstained with either fluorochrome) from apoptotic cells (stained only with Annexin V) and necrotic cells (stained with both Annexin V and propidium iodide).

### Flow-cytometric dye-efflux assay for multidrug resistance

The accumulation of rhodamine 123, a fluorescent substrate of P-gp, in CEM and CEM/VLB_100 _cells treated with or without TRAIL was measured using a FACS flowcytometer (FACScalibur, BD Biosciences, San Jose, CA) equipped with an ultraviolet argon laser (excitation at 488 nm and emission at 530 ± 15 nm). Cell suspension (500 μl) from CEM and CEM/VLB_100 _cells treated with or without 10 ng/ml TRAIL for 6 h was incubated with rhodamine 123 (0.5 μg/ml) at 37°C for 30 min. After incubation, the cells were washed with ice-cold PBS and further incubated at 37°C for 3 h to allow P-gp-mediated drug efflux or on ice (4°C) as control. Cells were pelleted by centrifugation at 500 × g and resuspended in PBS containing. Cellular fluorescence was analyzed immediately by using Flow cytometer.

### Statistical analysis

The results obtained were expressed as the mean ± S.E. of at least three independent experiments. The statistical significance of differences assessed using the Student's t-test. *p < 0.05, **p < 0.01, ***p < 0.005 was considered statistically significant in all experiments.

## Abbreviations

P-GP: P-GlycoProtein, tumor necrosis factor-related apoptosis-inducing ligand; TRAIL: C-FLIP, cellular FLICE inhibitory protein; MDR: multidrug resistance; PI3K: phosphoinositide 3-kinase; PAKT: DNA-PKcs, DNA-dependent protein kinase catalytic subunit, phosphorylated Akt, tAkt, total Akt; GSK-3α/β: glycogen synthase kinase-3α/β; PGSK-3β: phosphorylated GSK-3β, siRNA: small interfering RNA; CEM: human lymphoblastic leukemia; VCR: vincristine: VLB: vinblastine; DOX: doxorubicin.

## Competing interests

The authors declare that they have no competing interests.

## Authors' contributions

SBS, JGH, MJK, JWL, HBK and JHB designed and conducted experiments as well data analysis. DWK participated in discussion of the data and draft of the manuscript. SHK and CD K equally participated in experimental design, coordination, data analysis and draft of the manuscript. All authors read and approved the final manuscript.
